# Acute response of biomarkers in plasma from capillary blood after a strenuous endurance exercise bout

**DOI:** 10.1007/s00421-022-05068-1

**Published:** 2022-10-13

**Authors:** Thomas Reichel, Steffen Held, Anthony Schwarz, Sebastian Hacker, Fabian Wesemann, Lars Donath, Karsten Krüger

**Affiliations:** 1 Department of Exercise Physiology and Sports Therapy, Institute of Sports Science, Justus-Liebig University Giessen, Kugelberg 62, 35394 Giessen, Germany; 2 Intervention Research in Exercise Training, German Sports University, Cologne, Germany

**Keywords:** Biomarkers, Athletes, Training monitoring, Earlobe blood, Point-of-care testing

## Abstract

**Purpose:**

The present study aims to investigate the acute response of potential exercise-sensitive biomarkers in capillary plasma to an acute incremental running test. In a second step, their concentration was compared to the changes in the venous serum.

**Methods:**

Thirty-seven active young female and male adults completed a VO_2max_ ramp test on a treadmill. Before and after exercise, capillary blood from the earlobe and venous blood were taken and synchronized. Concentrations of Interleukin- (IL-) 1β, IL-1ra, IL-6, IL-8, IL-17A, Interferon (IFN)-y, CC-chemokine ligand (CCL)-2, Matrix metallopeptidase (MMP)-9, Secreted protein acidic and rich in cysteine (SPARC), Cluster of differentiation (CD)163, S100 Ca^2+^ -binding protein (S100) A8, S100A9, S100B, Brain-derived neurotrophic factor (BDNF), and Myeloperoxidase (MPO) were determined by magnetic bead-based multiplex assay.

**Results:**

Capillary plasma concentrations of IL-1β, IL-6, IL-8, IL-17A, IFN-y, CCL-2, MMP-9, SPARC, CD163, S100A9, S100B, and BDNF increased after exercise (*p* < 0.05). Comparing the values from capillary plasma and venous serum, ICCs classified as good were found for IFN-y (post), while the ICCs for IL-1β, IL-8, IL-17A, CCL-2, MMP-9 (post), SPARC, and BDNF (post) were classified as moderate. For all other parameters, only weak ICCs were found.

**Conclusion:**

As in the venous serum, there was an increase in most markers in the capillary plasma. However, acceptable to low associations can be found in the concentration levels of these proteins between the compartments. Thus, this source of blood sampling could find some biomarker applications in sports practice.

## Introduction

Today, specific diagnostic monitoring for athletes in elite sports is possible to individually control training load and ensure an optimized recovery (Meeusen et al. 2013; Barnett 2006). Scientific methods and techniques have been constantly improved to cover the high demand among users in elite sport to test, establish and then regularly use innovative parameters and tools for the diagnosis of stress and recovery cycles. Modern methods are also being used to reflect the athlete’s internal load more closely and accurately. Besides subjective recording via questionnaires, also various objective parameters, such as heart rate variability, cardiovascular parameters, functional measurements, and blood-based biomarkers are frequently used (Djaoui et al. 2017; Pickering and Kiely 2019; Wahl et al. 2021).

Blood-based markers, such as myokines, enzymes, growth factors, cytokines and other inflammatory signaling molecules have enormous development potential, as research is gaining increasing knowledge about their physiological background, the link to tissues being stressed, and their regulation pattern in blood (Banfi et al. 2012; Lee et al. 2017; Krüger et al. 2019). At the same time, biochemical measurement methods are becoming more sensitive, mobile, and inexpensive, so that the techniques can also be used by non-specialized staff. Thus, the aim for athletes and coaches should be to create a combination of efficient and convenient sample collection (Carling et al. 2018) as well as innovative point-of-care (POC) devices, mobile tools or microarray-based screenings (Jung et al. 2015; Sauer 2017). This avoids placing excessive burden for athletes and ensures reliable data through repeatable sample collection. Practically, this would mean that blood should not necessarily be obtained from the arm veins, but as plasma from capillary blood from the fingerpick or the earlobe (Siart et al. 2019). Many athletes are familiar with blood sampling from the earlobe, as this is also where lactate measurements are taken (Faulkner et al. 2014; Martinez-Navarro et al. 2021). In addition, taking the blood plasma from this source is more user-friendly, because it represents an easy collection up to 200 uL by a minimally invasive procedure and does not require skilled personnel (Raa et al. 2020). Accordingly, this method is also interesting for the application in competitive sports.

An important question remains to what extent intended blood markers can also be found in comparable concentrations to venous serum before and after exhaustive exercise. First of all, we and other scientists were able to identify several markers in human serum which demonstrated a certain suitability and reliability as biomarkers for exercise response and stress-recovery cycles. These markers include cytokines, such as interleukin (IL-)1β, IL-1 receptor antagonist, IL-6, IL-8, IL-17A, and Interferon gamma (IFN-γ), chemokines, such as CC-chemokine ligand 2 (CCL-2), matrix metallopeptidase (MMP), such as MMP-9, matrix and membrane proteins, such as secreted protein acidic and rich in cysteine (SPARC), and Cluster of differentiation (CD163), Ca^2+^-binding proteins, such as S100A8, S100A9, and S100B, growth factors, such as Brain-derived neurotrophic factor (BDNF), and enzymes such as myeloperoxidase (MPO) (Nieman et al. 2018; Reichel et al. 2020; Hacker et al. 2021). Concerning possible concentration differences between serum and plasma a closer examination using Bland–Altman analyses revealed that for some cytokines such as IL-1β, IL-6, and IL-8 the agreement was moderately good between both media (Parkitny et al. 2013). Regarding the concentrations of other cytokines, chemokines, or enzymes in plasma from capillary blood of the earlobe, there are only few data available. Most studies in this field addressed blood from the fingertip, which showed quite high similarities with venous blood for some markers (Keevil et al. 2009; Osteresch et al. 2016). Since we use venous serum as reference and plasma from capillary blood as comparison, immunological deviations could be expected (Parkitny et al. 2013; Siart et al. 2019).

In a first step, we aimed to quantify changes in plasma concentrations of several cytokines, chemokines, enzymes, and growth factors, in the capillary blood taken from the earlobe before and after an acute exercise bout. Here we focused on markers that have been shown to be exercise-sensitive (Reichel et al. 2020) and for which there is little data from capillary blood of the ear. In a second step, these changes were compared to the serum concentrations from venous blood taken from the arm veins. We hypothesized that most markers show a certain exercise-sensitivity in capillary blood. While there are certainly similarities between the levels in venous serum and capillary plasma for some markers, the exercise-effect will differ markedly for others.

## Methods

### Participants

Thirty-seven active young female and male adults were enrolled in this study (Table 1). All participants were at least 18 years of age and had no health impairments after medical examination. The study protocol complied with the Declaration of Helsinki, was approved by the local ethical committee (009/2021—Ethics Commission, German Sport University, Cologne), and fulfilled the international ethical standards (Harriss and Atkinson 2015). All participants signed an informed consent after receiving all relevant study information.

**Table 1:** Anthropometric data

	Female (*n* = 12)	Male (*n* = 25)	Total (*n* = 37)
Age (years)	23.8 ± 1.2	23.6 ± 4.6	23.7 ± 3.8
Height (m)	1.69 ± 0.05	1.82 ± 0.07	1.77 ± 0.09
Body mass (kg)	64.0 ± 5.5	77.2 ± 9.3	72.9 ± 10.3
Body fat (%)	20.0 ± 3.6	11.9 ± 3.7	14.6 ± 5.3
VO_2max_ (ml/min/kg)	50 ± 7	60 ± 7	56 ± 9

Number of subjects (*n*), Means ± standard deviation

### Study design

Using a pre-post design, subjects completed an incremental ramp test on a treadmill. At the beginning of the test procedure, a standardized 2-min warm-up was completed at low intensity (blood lactate < 2 mmol/L). VO_2max_ was determined via a ramp test to exhaustion on a treadmill (PPS Med treadmill, Woodway, Waukesha USA): start at 2.4 m/s followed by an increment from 0.2 m/s per 30 s to 4 m/s; thereafter 0.5% incline increment per 30 s. The VO_2max_ as well as the objective exhaustion of the athletes was verified according to the guidelines established by Midgley et al. (2007) for all participants. Fifteen minutes before and immediately after the VO_2max_ test, blood was taken. This sampling procedure is described in the following section. The first blood sample was taken after a 10-min rest period. Subsequently, each subject received an individualized fixed amount of carbohydrates (0.5 g maltodextrin/kg body mass) to ensure standardized conditions. This was to minimize interindividual variability in food intake in terms of time and content prior to the ramp test. In addition, all subjects were familiarized with the testing procedure before the start of the study and motivated in the same way at the end of the testing procedures. Participants were instructed to avoid any strenuous physical exertion two days prior to the diagnostic appointment and attended without having their regular breakfast.

### Blood sampling and pre-analytic

3–5 ml venous blood samples were taken from a vein in the bend of the arm using S-serum monovettes. In addition, approximately 200 μL of capillary blood were taken from the earlobe by plasma microvettes. To achieve better blood supply, the earlobes were rubbed with a skin-warming gel (Finalgon—active ingredients nonivamide and nicoboxil). Venous samples were initially stored in an upright position for 15 min after sampling. This was followed by centrifugation at 2000 times gravity and 4 °C for 15 min to separate the blood into serum and cellular fractions. After the respective aliquoting of plasma and serum using a pipette, samples were quickly frozen at – 80 °C.

### Biomarker analysis

Plasma and serum levels of IL-1β (Analytical sensitivity (AS): 0.8 pg/mL; Coefficient of variation (CV): 2.3%), IL-1ra (AS: 18.0 pg/mL; CV: 2.1%), IL-6 (AS: 1.7 pg/mL; CV: 2.6%), IL-8 (AS: 1.8 pg/mL; CV: 1.8%), IL-17A (AS: 1.8 pg/mL; CV: 2.5%), IFN-y (AS: 0.4 pg/mL; CV: 2.8%), CCL-2 (AS: 9.9 pg/mL; CV: 1.4%), MMP-9 (AS: 13.6 pg/mL), SPARC (AS: 97.9 pg/mL), CD163 (AS: 530 pg/mL), S100A8 (AS: 15.3 pg/mL), S100A9 (AS: 6.39 pg/mL), S100B (AS: 4.34 pg/mL), BDNF (AS: 0.32 pg/mL), and MPO (AS: 26.2 pg/mL) were determined using a human Magnetic Luminex Assay (Bio-Techne, Abingdon, Oxon, UK) using a Magpix Luminex instrument with valid xMax technology (Luminex Corp, Austin, Texas, US) (Satterly et al. 2016). The Magnetic Luminex assays were pre-coated with analyte-specific antibodies on magnetic microparticles embedded with fluorophores in specific ratios for each individual microparticle region. 50 µL Microparticles, 50 µL standards and 50 µL samples were pipetted into the wells and the immobilized antibodies bound the analytes of interest. After incubation on a shaker for two hours and washing off all unbound substances, a biotinylated antibody cocktail of 50 µL was added to the analytes in each well. After another one-hour incubation and another wash to remove unbound biotinylated antibody, a streptavidin–phycoerythrin conjugate (streptavidin-PE) of 50 µL was added to each well to bind to the biotinylated antibody. Final washing after a 30-min incubation removed unbound streptavidin-PE, the microparticles were resuspended in 100 µL buffer and analyzed with the Magpix Luminex instrument.

### Statistics

All data are presented as group means ± standard deviation or with 95% confidence intervals. First, the data that fell out of the analytical range due to insufficient measurement sensitivity were omitted. After that the data were cleaned for outliers using *z*-transformation (*z*-score > 3 standard deviations) (Stocker and Steinke 2016). Accordingly, differentiated N-values were formed. Data were visually inspected for normal distribution and variance homogeneity. Further, the data were analyzed using the pairwise *T* test for normal distributed data and Wilcoxon-Test for non-normal distributed data. A *p* value of ≤ 0.05 was accepted as statistically significant. As the study corresponds to an exploratory approach, there was no multiple test adjustment of the α-error. Standardized mean differences (SMD; trivial: SMD <|0.2|; small: |0.2|≤ SMD <|0.5|; moderate: |0.5|≤ SMD <|0.8|; large SMD ≥|0.8|), effect size, and rank-biserial correlation (*r*; small: |0.1|≤ *r* <|0.3|; moderate: |0.3|≤ *r* <|0.5|; large *r* ≥|0.5|) as a measure of the pairwise effect size estimation were also calculated (Cohen 1988). The agreement between both blood withdrawal locations (venous vs. capillary) were analyzed by calculating the systematic bias (mean difference between devices) and the limits of agreement (LoA: 1.96*standard deviation of the difference between both devices), considering a 95% random error component (Atkinson and Nevill 1998) and plotting several Bland–Altman plots (Bland and Altman 1986). Intraclass correlation coefficients (ICC) were additionally calculated (Atkinson and Nevill 1998). ICC were rated as excellent (0.9–1), good (0.75–0.9), moderate (0.5–0.75) and poor (0–0.5) (Koo and Li 2016). A linear regression analyses was calculated to analyze associations based on the dependent venous blood values on the independent capillary blood values. Thereby, regression coefficient (*B*), standard error of regression coefficient (SEB), intercept, coefficient of determination (*R*), adjusted multiple coefficient of determination (*R*^2^), and significance level (*p*) are given (Cohen 1988). The statistical analyses were conducted using SPSS version 26 (IBM SPSS Statistics 26, IBM GmbH, Munich, Germany). The Bland–Altman plots were created using the logarithmised values for better comparability of the biomarkers (Prism 9, GraphPad Software, San Diego, CA, USA).

## Results

### Effects of exercise on markers in plasma from capillary blood

**Fig. 1:**
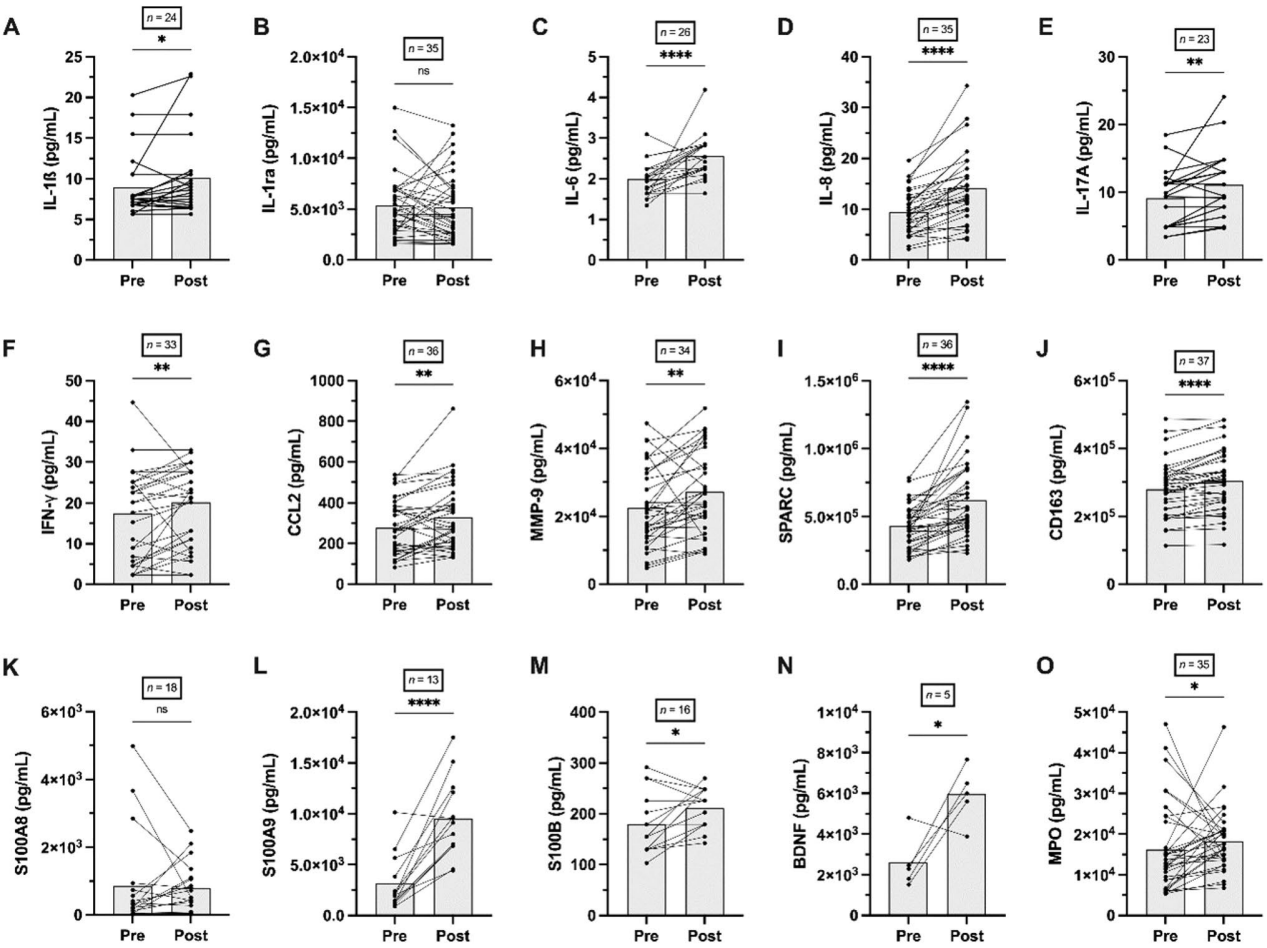
Changes of the concentration of various cytokines, chemokines, matrix metallopeptidases, membrane proteins, Ca^2+^-binding proteins, growth factors, and enzymes pre and post-acute exercise in capillary plasma taken from the earlobe. Number of subjects (*n*), results from the pairwise *T* test as well as Wilcoxon-Test: *mean *p* < 0.05, **means *p* < 0.01, ****means *p* < 0.0001. Raw data are shown

Significant increases during exercise in plasma concentrations were found for IL-1β (*p* = 0.022; *d* =  – 0.57; *r* = – 0.63), IL-6 (*p* < 0.001; *d* =  – 0.89; *r* =  – 0.89), IL-8 (*p* < 0.001; *d* =  – 0.98; SMD = 1.38), IL-17A (*p* = 0.006; *d* =  – 0.66; SMD = 0.63), IFN-y (*p* = 0.002; *d* =  – 0.61; *r* =  – 0.60), CCL-2 (*p* = 0.003; *d* =  – 0.55; SMD = 0.53), MMP-9 (*p* = 0.005; *d* =  – 0.50; SMD = 0.51), SPARC (*p* < 0.001; *d* =  – 0.93; SMD = 1.12), CD163 (*p* < 0.001; *d* =  – 0.80; SMD = 0.90), S100A9 (*p* < 0.001; *d* =  – 0.97; SMD = 1.54), S100B (*p* = 0.017; *d* =  – 0.67; SMD = 0.62), BDNF (*p* = 0.038; *d* =  – 1.35; SMD = 1.04), while no changes were detected in plasma levels of IL-1ra, MPO, and S100A8 (*p* > 0.05) (Fig. 1). The effects of exercise on markers in serum from venous blood can be found in Table 2.

**Table 2:** Changes of the concentration of various blood-based markers pre and post-acute exercise in venous serum

Measure 1	Measure 2	Test	*p*	*d*
IL-1β PRE	IL-1β POST	Wilcoxon-Test	**0.005**	** – **0.66
IL-1ra PRE	IL-1ra POST	Wilcoxon-Test	** < 0.001**	** – **0.85
IL-6 PRE	IL-6 POST	Wilcoxon-Test	** < 0.001**	** – **0.85
IL-8 PRE	IL-8 POST	Pairwaise T-Test	** < 0.001**	** – **0.68
IL-17a PRE	IL-17a POST	Wilcoxon-Test	0.640	** – **0.14
IFN-γ PRE	IFN-γ POST	Pairwaise T-Test	**0.002**	** – **0.80
CCL2 PRE	CCL2 POST	Wilcoxon-Test	** < 0.001**	** – **0.79
MMP-9 PRE	MMP-9 POST	Wilcoxon-Test	** < 0.001**	** – **0.95
SPARC PRE	SPARC POST	Wilcoxon-Test	** < 0.001**	** – **1.00
CD163 PRE	CD163 POST	Wilcoxon-Test	** < 0.001**	** – **0.82
S100A8 PRE	S100A8 POST	Wilcoxon-Test	**0.003**	** – **0.90
S100A9 PRE	S100A9 POST	Wilcoxon-Test	** < 0.001**	** – **1.00
S100B PRE	S100B POST	Pairwaise T-Test	**0.003**	** – **0.82
BDNF PRE	BDNF POST	Pairwaise T-Test	0.789	** – **0.10
MPO PRE	MPO POST	Wilcoxon-Test	** < 0.001**	** – **0.69

Significance (*p*), Effect size according Cohen’s (*d*), Highlighted are significant *p* values < 0.05

### Agreement between the marker regulation in capillary plasma and venous serum

**Fig. 2:**
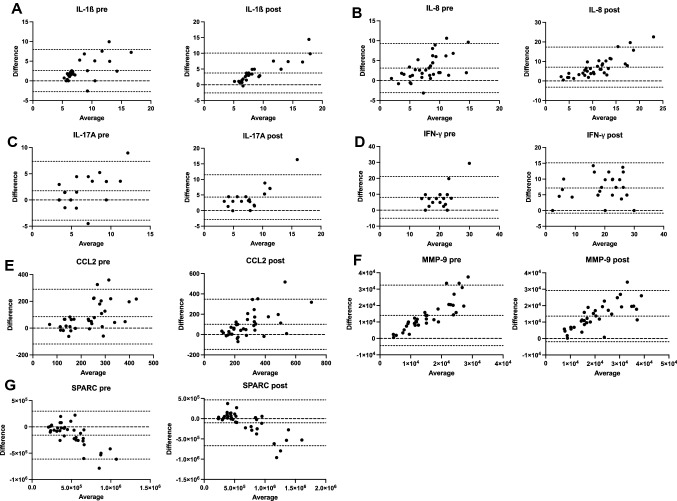
Bland–Altman plots for IL-1β (**A**), IL-8 (**B**), IL-17A (**C**), IFN-y (**D**), CCL-2 (**E**), MMP-9 (**F**), and SPARC (**G**). Raw data are shown. PRE samples taken before exercise; POST samples taken after exercise

Table 3 presents the intraclass correlations (ICCs) and 95% confidence intervals (CIs) depicting the agreement between the marker concentration in capillary plasma and venous serum. While IFN-y (post) showed a good ICC-value (ICC = 0.87), the ICCs of IL-1β, IL-8, IL-17A, CCL-2, MMP-9 (post), SPARC, and BDNF (post) can be classified as moderate (0.5–0.75; Table 3). For all other time points and parameters, ICCs were classified as poor (< 0.5; Table 3). Table 3 shows higher venous concentrations compared to capillary blood for negative bias, and higher capillary blood concentrations compared to venous blood for positive values. In general, IL-1ß, IL-8, IL-17A, CCL-2, MMP-9 (post), SPARC, and BNDF (post) show a good to moderate agreement for both compartments and results as valid markers. The regression data support these results, since for IFN-y, IL-1ß, IL-8, IL-17a, CCL-2, MMP-9 (post), and SPARC (Table 4) significant associations with results of maximum low adjusted coefficient of determination (*R*^2^) > 0.3 were shown. This indicates that the variance of the previously mentioned biomarker concentrations in capillary plasma can be predicted to 30–82% by the concentration of the markers in venous serum. BA plots showed acceptable LoA’s between the two compartments for IFN-y (post), IL-8, and IL-17A (Fig. 2). However, this doesn’t exclude the markers IL-1ß, CCL-2, MMP-9 (post) and SPARC in their validation, but in their practicability (Fig. 2). All other markers with a low agreement and a higher scattered distribution are not shown in Fig. 2.

**Table 3:** Evaluations of the agreement of venous and capillary measurements using Intraclass correlation coefficient and Bland–Altman analysis

Parameter	Time	*N*	Bias [pg/mL]	SD of bias [pg/mL]	Limits of agreement [pg/mL]	ICC (95% CI)
IL-1β	PRE	28	2.62	2.74	5.37	0.65 (0.38–0.82)*
	POST	28	3.72	3.23	6.32	0.68 (0.43–0.84)*
IL-1ra	PRE	34	5149	3171	6215.16	< 0.0 (**– **0.34–0.32)
	POST	36	5017	3382	6628.72	< 0.0 (-0.33–0.31)
IL-6	PRE	23	0.65	0.62	1.21	0.00 (**– **0.40–0.40)
	POST	34	0.9	0.55	1.07	0.23 (**– **0.12–0.54)
IL-8	PRE	35	3.12	3.14	6.15	0.56 (0.28–0.75)*
	POST	36	7.07	5.23	10.24	0.49 (0.19–0.70)*
IL-17a	PRE	22	1.75	2.86	5.60	0.56 (0.19–0.79)*
	POST	20	4.31	3.66	7.16	0.48 (0.05–0.75)*
IFN-γ	PRE	20	8.03	6.67	13.06	0.11 (**– **0.33–0.51)
	POST	26	7.14	4.08	7.99	0.87 (0.73–0.94)**
CCL2	PRE	35	85.66	104.1	204.03	0.48 (0.19–0.70)
	POST	37	100.6	126.1	247.15	0.62 (0.38–0.78)*
MMP-9	PRE	35	14,019	9387	18,398.52	0.39 (0.08–0.64)
	POST	35	13,605	7950	15,582	0.69 (0.47–0.83)*
SPARC	PRE	36	** – **157,887	232,925	456,533	0.57 (0.30–0.75)*
	POST	36	** – **103,981	288,590	565,636.4	0.72 (0.52–0.85)*
CD163	PRE	37	** – **301,683	366,253	717,855.88	0.23 (**– **0.09–0.51)
	POST	37	** – **389,287	465,762	912,893.52	0.21 (**– **0.11–0.50)
S100A8	PRE	17	908.5	1463	2867.48	0.00 (**– **0.46–0.47)
	POST	16	837.9	756.2	1482.15	0.00 (**– **0.48–0.48)
S100A9	PRE	15	2149	2589	5074.44	0.05 (**– **0.45–0.53)
	POST	16	6966	3539	6936.44	0.22 (-0.29–0.63)
S100B	PRE	17	** – **32.14	73.33	143.72	0.07 (**– **0.40–0.52)
	POST	17	** – **65.17	65.04	127.47	0.12 (**– **0.36–0.56)
BDNF	PRE	9	** – **1382	2398	4700.08	0.26 (**– **0.43–0.76)
	POST	5	93.96	1631	3196.76	0.55 (**– **0.47–0.94)*
MPO	PRE	35	2681	10,720	21,011.2	0.12 (**– **0.21–0.43)
	POST	35	2415	7669	15,031.24	0.42 (0.10–0.66)

For negative bias the concentrations in venous blood are higher than in capillary blood, whereas for positive bias the concentration in capillary blood are higher than in venous blood

*N* Number of subjects, *SD* standard deviation of bias, *ICC* Intraclass correlation coefficient, *CI* Confidence interval (CI), No * means poor ICC, * means moderate ICC, ** means good ICC

**Table 4:** Prognostic association values between venous and capillary blood data using linear regression

Parameter	Time	*N*	*B* [pg/ml]	Intercept [pg/ml]	SEB [pg/ml]	*R*	*R*^2^	*p*
IL-1ß	PRE	28	1.243	0.93	0.21	0.746	0.557	** < 0.001**
	POST	28	1.980	** – **3.34	0.17	0.908	0.825	** < 0.001**
	Δ	30	1.083	3.31	1.25	0.161	0.026	0.396
IL-1ra	PRE	34	** – **13.324	8086.77	7.33	0.306	0.094	0.079
	POST	36	** – **6.952	7503.27	5.49	0.212	0.045	0.214
	Δ	34	4.245	** – **400.21	10.53	0.071	0.005	0.690
IL-6	PRE	23	0.005	1.96	0.18	0.005	< 0.001	0.980
	POST	30	0.256	2.12	0.19	0.238	0.057	0.205
	Δ	20	0.822	0.30	0.43	0.407	0.165	0.075
IL-8	PRE	35	1.042	2.84	0.22	0.635	0.403	** < 0.001**
	POST	36	1.786	1.25	0.30	0.712	0.507	** < 0.001**
	Δ	35	** – **0.082	4.77	0.48	0.030	0.001	0.866
IL-17a	PRE	22	1.041	1.52	0.28	0.632	0.399	**0.002**
	POST	20	1.471	1.60	0.40	0.649	0.421	**0.002**
	Δ	13	0.021	1.38	0.43	0.015	< 0.001	0.962
IFN-γ	PRE	20	0.276	19.70	0.46	0.139	0.019	0.559
	POST	26	0.936	8.14	0.10	0.874	0.764	** < 0.001**
	Δ	20	0.434	0.99	0.32	0.300	0.090	0.199
CCL2	PRE	35	0.919	100.82	0.24	0.554	0.306	** < 0.001**
	POST	37	1.148	64.98	0.19	0.704	0.495	** < 0.001**
	Δ	35	** – **0.085	43.81	0.23	0.062	0.004	0.722
MMP-9	PRE	35	1.960	5660.87	0.38	0.660	0.436	** < 0.001**
	POST	35	1.477	6815.39	0.18	0.817	0.668	** < 0.001**
	Δ	34	1.046	** – **1088.96	0.33	0.479	0.230	**0.004**
SPARC	PRE	36	0.352	222,671.06	0.05	0.730	0.532	** < 0.001**
	POST	36	0.474	275,786.45	0.04	0.861	0.741	** < 0.001**
	Δ	36	0.474	138,096.91	0.16	0.452	0.205	**0.006**
CD163	PRE	37	0.122	209,132.08	0.02	0.623	0.388	** < 0.001**
	POST	37	0.112	226,891.25	0.02	0.657	0.431	** < 0.001**
	Δ	37	0.077	15,450.01	0.02	0.469	0.220	**0.003**
S100A8	PRE	17	61.479	445.19	60.45	0.254	0.064	0.325
	POST	16	6.757	760.50	25.79	0.070	0.005	0.797
	Δ	16	64.135	** – **486.78	49.32	0.328	0.108	0.215
S100A9	PRE	15	1.501	1789.60	2.03	0.200	0.040	0.474
	POST	16	0.536	8209.38	0.50	0.274	0.075	0.305
	Δ	13	0.314	5865.49	0.84	0.112	0.012	0.716
S100B	PRE	17	0.089	162.61	0.28	0.080	0.006	0.762
	POST	17	0.087	189.36	0.16	0.138	0.019	0.597
	Δ	16	** – **0.063	36.60	0.16	0.101	0.010	0.711
BDNF	PRE	9	0.306	2270.08	0.41	0.269	0.073	0.484
	POST	5	0.448	3459.60	0.37	0.565	0.319	0.321
	Δ	5	0.070	4002.44	0.61	0.065	0.004	0.917
MPO	PRE	35	0.432	10,446.58	0.41	0.179	0.032	0.305
	POST	35	0.456	11,070.13	0.16	0.424	0.180	**0.011**
	Δ	34	0.548	380.22	0.49	0.192	0.037	0.277

*N* Number of subjects, *B* regression coefficient, *SEB* standard error of regression coefficient, *R* intercept, coefficient of determination, *R*^2^ adjusted coefficient of determination, *p* significance, *Δ* pre-post delta values

Highlighted are significant *p* values < 0.05

## Discussion

The aim of the present study was to investigate the response of biomarkers in capillary blood after a strenuous endurance exercise bout. Further, we compared the concentration between the compartments to analyze agreements or a similar increase after exercise. The study demonstrated that several blood markers, which were selected based on their significance as potential biomarkers of the exercise stress-response, are detectable in capillary blood taken from the earlobe before and after an acute exercise test. As expected, IL-1β, IL-6, IL-8, IL-17A, IFN-y, CCL-2, MMP-9, SPARC, CD163, S100A9, S100B, and BDNF showed a significant increase after the acute exercise protocol, indicating their suitability to reflect the acute exercise response. However, compared to serum taken from arm veins, concentrations and the regulation after exercise shows significant heterogeneities. Except for IFN-γ (ICC: good), most other parameters show moderate to poor ICCs before and after exercise. The agreement between the concentrations in both compartments is higher for IL-1β, IL-8, IL-17A, CCL-2, MMP-9, and SPARC before and after exercise, lower agreements were found for all other markers. However, the results for the markers IFN-y, IL-8, and IL-17A indicate an acceptable agreement between concentration values in capillary plasma and venous serum.

The profiles for the analyzed markers in the context of exercise stress have not been well studied, which means that there are no reference values for most of the selected markers (Monastero and Pentyala 2017). Accordingly, it is difficult to classify absolute values, specifically the deviating values in capillary plasma. First, it was important for us to show that the selected markers also increase in capillary blood. It is thus clear that, like our previous data from venous serum, they can also indicate an acute exercise response here. The lack of increase in IL-1ra, S100B and MPO can certainly be explained. IL-1ra secretion into blood is induced after a short delay after exercise, so that significant increases were often seen in the period of three hours after exercise (Reichel et al. 2020). Such regulation was not expected immediately after an incremental running test. For S100B and MPO, we assume that the extent and intensity of the stress applied here was not sufficient. In previous studies, the increase of these specific markers was shown after marathon runs or intensive and prolonged exercise tests. For both markers, it was shown here that their regulation is intensity-dependent and related to exercise induced muscle damage (Camus et al. 1992; Mooren et al. 2006).

In the next step, we performed similarities to serum concentrations from the brachial vein. The protocol made it possible to obtain both samples almost synchronously. First, it must be said that we consistently found higher deviations in cytokine concentrations between the two compartments than initially assumed. We will discuss how deviations across all markers could be physiologically justified. Both blood compartments certainly differ first in their saturation with respiratory gases since capillary blood is arterial-venous mixed blood (Zavorsky et al. 2007). Hematocrit and hemoglobin concentration have been found to be higher in capillary samples (Daae et al. 1988), while for the number of leukocytes were found conflicting results (Schalk et al. 2007; Canetti et al. 2016). However, it was repeatedly demonstrated that there are functional differences in immune cells between the two compartments, especially in the function of granulocytes and monocytes (Canetti et al. 2016). Some of the marked differences in the regulation of IL-1β, CCL-2, IL-1ra, IL-8, and MMP-9 may be due to this, as these cytokines are produced by activated neutrophils and monocytes. If the number of cells per time unit in these compartments differs only slightly, this can lead to significant differences in cytokine concentrations (Webster and Crowe 2006). However, the consistently higher values of IL-1ra in capillary plasma, both before and after exercise can certainly not be completely explained in this way. Hence, other mechanisms are probably active here. We assume that these large differences in concentration can occur within the framework of the coagulation process during serum formation. The S-monovettes contain granules coated with a clot activator (silicate). The coagulation process could stimulate an increased secretion of specific cytokines (Johnson et al. 1996; Van der Poll et al. 2001). This explanation also applies to cytokines such as IL-1β, IL-6, and IL-10, which secretion is also stimulated in the coagulation process (van der Poll et al. 2001).

For IFN-γ, a high agreement between the compartments was found after exercise. On the one hand, this shows that the cytokine is little influenced by the coagulation process. On the other hand, it is noticeable that the values after exercise show a higher agreement compared to pre-exercise. This phenomenon was also seen with other parameters, such as IFN-y, CCL-2, MMP-9, SPARC, and BDNF. We assume that measurement sensitivity improves in the middle to high measurement range as we move further away from the lower detection limit.

We further suggest that a local immune stimulation has taken place through the extraction of blood from the ear and the associated tissue injury. Luminex analysis required the measurement in 50 μl plasma, for which a total of 200 μl whole blood had to be taken from the ear. To do this, the earlobe was cut open with a lancet and blood was also obtained under a little pressure on the earlobe. On the one hand this could have led to an immunological reaction, which resulted in an activation of the tissue cells and leukocytes (Furie and Randolph 1995). On the other hand, this could also mean that more lymph was squeezed out, whereby cytokines, which are present in different concentrations in the lymph fluid, affected the blood values (Aldrich and Sevick-Muraca 2013). This applies, for example, to IL-1β and IL-6, for which it has been shown that they are present in higher concentrations in the lymph fluid (Aldrich and Sevick-Muraca 2013).

Cytokine release could be caused by pre-treatment of the ear with nonivamide and nicoboxil. These substances are known to improve blood flow to the ear capillaries and facilitate blood collection (Moro et al. 2017). As the blood had to be obtained quickly in sync with the vein collection, this pre-treatment was necessary. Nicotoxil has a vasodilatory effect, resulting in increased skin blood flow and local heating. Nonivamide penetrates the skin tissue and docks here at special binding sites of the nerve cells, whereby analgesic messenger substances are released (Stücker et al. 1999; Hoekstra et al. 2020). While data show that there is little effect on leukocytes from pre-treatment, we can only speculate at the effects on cytokines (Moro et al. 2017). However, cayenne pepper thick extract, which is often added to such active substances, was not present in this preparation.

SPARC and IL-17A show a moderate agreement between the compartments. SPARC is a matrix-associated glycoprotein which is secreted by osteoblasts during bone formation. The contracting skeletal muscle has recently been identified as the source of SPARC, which means that SPARC must also be classified as a myokine (Aoi et al. 2013). It binds to several proteins of the extracellular matrix (ECM), affect ECM protein expression, and modulate growth factor-induced cell proliferation and angiogenesis. We suggest that SPARC secretion is not strongly affected by blood sampling, as SPARC is not primarily released by tissue injury and leukocyte activity. In addition, SPARC is not secreted by muscle cells which might explain the slight deviation between the compartments after exercise (Phan et al. 2007). The acceptable concordance of IL-17A in both compartments may be because it is primarily produced by T cells, which are probably very similarly concentrated in both compartments (Sugama et al. 2012).

The low ICC of IL-6 and moderate ICC of IL-8 between both compartments could additionally be explained by their classification as myokines. Both interleukins are also released into the circulation by the contracting muscle (Della Gatta et al. 2014; Peake et al. 2015; Hojman et al. 2019). Due the different connection of the compartments to blood vessels leaving the muscle this might contribute to the different concentrations of these cytokines, especially after exercise.

Accordingly, markers secreted by a few cell types or from clearly defined sources seem to show less variation. The poor ICCs and agreements on S100A8/A9 seem to have different causes. On the one hand, these proteins show very high interindividual variations, probably because they are highly sensitive regulated. Furthermore, both markers are much more concentrated in capillary plasma than in venous blood, which can also be attributed to a significant effect of the coagulation process. Both proteins are constitutively expressed in neutrophils and monocytes as a Ca^2+^ sensor, participating in cytoskeleton rearrangement and arachidonic acid metabolism. In the case of an inflammatory stimulus, they rise very immediately and quickly (Wang et al. 2018).

Finally, a few limitations need to be discussed. First of all, an incremental running test, although regularly used in performance diagnostics, is not used in athletic training or even many studies. The type of capillary blood collection from the earlobe is also controversial. Getting 200 µl of capillary blood out of the ear requires good handling and can leave a hematoma on the ear. Excessive massaging or squeezing of the puncture site should be avoided to prevent hemolysis, contamination of the blood with interstitial and intracellular fluid, and obstruction of blood flow (Godfrey et al. 2004). For some markers, such as BDNF, we had difficulty with sensitivity. We did not allow interpolation of values and proceeded conservatively, so we still want to show them with transparent presentation of limitations.

The present study shows that the regulation of possible biomarkers in sport shows clear differences between capillary blood and venous blood. Some markers show a higher, others a significantly lower agreement. Differences in the blood composition, the modality of tissue treatment in the context of blood collection, and the coagulation process seem to have a strong effect on the concentration especially on the inflammation-sensitive markers secreted by leukocytes.

### Practical implications

These data confirm the assumption that, especially in the case of cytokines without clear reference values, an individual range must first be established for each athlete, also considering the blood collection site. At the same time, the collection procedure must be strongly standardized. Future studies should investigate the extent to which these ranges in capillary blood can then be used to monitor the stress-recovery cycles of athletes. This type of blood sampling is a much more user-friendly, especially for athletes. Future studies should further validate the marker concentrations in capillary plasma and perform time series analyses to account for the individuality of the exercise response of each marker. A clinical application of the analyses in capillary blood is also conceivable, for example when it comes to vulnerable patient groups in whom cytokines are to be measured as non-invasively as possible.

## Data Availability

The datasets generated during and/or analyzed during the current study are available from the corresponding author on reasonable request.

## Abbreviations

BDNF: Brain-derived neurotrophic factor
CCL-2: CC-chemokine ligand 2
CD163: Cluster of differentiation 163
IFN-y: Interferon gamma
IL: Interleukin
MMP: Matrix metallopeptidase
MPO: Myeloperoxidase
SPARC: Secreted protein acidic and rich in cysteine
S100 A8/A9/B: S100 Ca^2+^-binding protein A8/A9/B
VO_2max_: Maximal oxygen consumption
